# A cross -sectional study on social media addiction and its association with sleep quality among young students of age group 18-25 in Madhya Pradesh

**DOI:** 10.6026/973206300220794

**Published:** 2026-02-28

**Authors:** Pravin Yerpude, Keerti Jogdand, Dileep Dandotiya, Sharad M Manore

**Affiliations:** 1 Department of Community Medicine, Chhindwara Institute of Medical Sciences, Chhindwara, Madhya Pradesh, India; 2 Department of Psychiatry, Chhindwara Institute of Medical Sciences, Chhindwara, Madhya Pradesh, India

**Keywords:** Social media addiction, sleep quality, Pittsburgh Sleep Quality Index (PSQI), Bergen Social Media Addiction Scale (BSMAS), students, cross-sectional study

## Abstract

The extensive use of social media by young people is causing worries about how it may affect their physical and mental well-being, especially their ability to sleep. Finding out how common social media addiction is and how it affects sleep quality among students in Chhindwara, Madhya Pradesh, between the ages of 18 and 25, was the goal of this study. The Pittsburgh Sleep Quality Index (PSQI) and the Bergen Social Media Addiction Scale (BSMAS) were used in a cross-sectional study involving 750 college students. Data on demographics and behaviour was gathered using a semi-structured questionnaire. 18.3% of participants were found to be addicted to social media. Higher BSMAS scores were substantially correlated with poorer sleep quality (r = 0.62, p < 0.001). Female gender, early morning usage and more than three hours of daily social media use were all factors that significantly predicted addiction. Those who used social media extensively, posted selfies frequently and experienced digital eye strain had the worst sleep quality. Addiction to social media is strongly linked to young students' poor sleep quality.

## Background:

The quick spread of smartphones with internet access and reasonably priced data plans over the last ten years has completely changed communication, especially among teenagers and young adults. Because social media platforms like facebook, instagram and whatsapp provide instant access to entertainment, information and social connections, they have become essential parts of everyday life. India has the third-largest user base in the world, with over 470 million active users as of 2023. The majority of daily users are between the ages of 18 and 25 [1]. Although social media makes networking and information sharing easier, excessive use of the platform has led to a phenomenon known as "social media addiction" a pattern of behaviour marked by excessive and compulsive engagement despite negative outcomes [2]. From a neurobiological perspective, this disorder shares mechanism with other types of behavioural addiction, such as the dopamine reward system being activated and repetitive behaviour is reinforced by "likes," shares and instant feedback loops [3]. The detrimental effects of excessive social media use on sleep quality are among the most alarming consequences. Excessive screen time, especially at night, has been linked in numerous studies to decreased sleep duration, delayed sleep onset and decreased sleep efficiency [4, 5]. Sleeplessness and daytime exhaustion are caused by the suppression of melatonin production and the disruption of the circadian rhythm caused by exposure to blue light from smartphones and tablets [6]. Sleep disturbances are also exacerbated by the cognitive stimulation brought on by emotionally charged or stressful content [7]. The poor quality of sleep in students is particularly worrying, as it is associated with poorer academic performance, poor mental health, poor concentration and an increased risk of depression and anxiety [8]. There is a strong correlation between high use of social media and poor sleep performance in adolescents and young adults, as reported in India and elsewhere [9]. In addition, the gender gap in social media use, with women generally showing greater emotional dependency and social comparison behaviour, may predispose them to both addiction and sleep deprivation [10]. Therefore, it is of interest to evaluate the social media addiction and its association with sleep quality among young students.

## Materials and Methods:

## Study design and setting:

Between February 2025 and April 2025, undergraduate students enrolled in different colleges in Chhindwara City, Madhya Pradesh, India, participated in this cross-sectional observational study. A growing centre for education, Chhindwara draws pupils from both urban and rural areas. The purpose of the study was to determine how common social media addiction is and how it affects sleep quality in a sample of young people between the ages of 18 and 25.

## Study population and sampling technique:

Students ages 18 to 25 that were enrolled in graduate programs in a variety of fields, including the arts, sciences, business and professional studies, made up the study population (e.g. G. BBA, BCA). Students were eligible for inclusion if they had been active social media users for at least two months before the data was collected.

## A multistage sampling technique was employed:

[1] Stage 1: Four-degree colleges were randomly selected from a list of registered colleges in Chhindwara.

[2] Stage 2: From each selected college, departments were randomly chosen.

[3] Stage 3: Within each department, students were selected using systematic random sampling from class attendance lists.

## Sample size estimation:

The minimum required sample size was determined to be 640 based on a 95 percent confidence interval, a 5 percent absolute precision, a 10 percent non-response rate and an expected prevalence of social media addiction of 20 percent. In the end, 750 students were enrolled to guarantee robustness and account for exclusions.

## Inclusion and exclusion criteria:

## Inclusion criteria:

[1] Students aged 18 to 25 years

[2] Active users of social media platforms for non-academic purposes

[3] Provided written informed consent

## Exclusion criteria:

[1] Students diagnosed with any psychiatric illness or on psychotropic medication

[2] Individuals with diagnosed sleep disorders or shift-working schedules

[3] Incomplete or inconsistent responses in the survey

## Data collection tools:

## The data collection instrument was a structured questionnaire composed of three parts:

[1] Socio-demographic profile: This section collected data on lifestyle factors, screen time habits, residence type (day scholar or hosteller), age, gender, course of study and year of study.

[2] Bergen Social Media Addiction Scale (BSMAS): This validated 6-item test assesses addiction to social media. The total score ranges from 6 to 30, with each item being scored on a 5-point Likert scale (1 being very rarely and 5 being very often). The diagnostic threshold for detecting social media addiction was set at ≥24. According to the fundamental elements of addiction, the scale assesses symptoms like salience, mood modification, tolerance, withdrawal, conflict and relapse.

[3] Pittsburgh Sleep Quality Index (PSQI): This popular 19-item self-report test evaluates the subjective quality of sleep during the past month. Subjective sleep quality, sleep latency, duration, habitual sleep efficiency, sleep disturbances, use of sleeping pills and dysfunction during the day are its seven constituents. The global PSQI score, which ranges from 0 to 21, is the sum of the component scores. Participants were categorized as having poor sleep quality if their global score was greater than 5.

## Data collection procedure:

Data were gathered in classroom settings by distributing printed questionnaires in person. Field investigators with training led each session, ensuring voluntary participation and providing students with an overview of the study's goals. The questionnaire was completed in 15-20 minutes on average. Forms were anonymized by employing distinct identifiers. Confidentiality was guaranteed and no faculty members were present during data collection in order to reduce social desirability bias. During data screening, any forms that were not complete were eliminated.

## Ethical considerations:

Institutional Ethics Committee ethical clearance was acquired. Each participant was made aware of the goals of the study, the fact that participation was entirely voluntary and their freedom to leave at any moment without incurring any fees. Prior to involvement, written informed consent was acquired. The Declaration of Helsinki's ethical principles were followed in this investigation.

## Statistical analysis:

IBM SPSS version 25 was used for analysis after all responses were coded and imported into Microsoft Excel. Sociodemographic variables were subjected to descriptive statistics. Poor sleep quality and social media addiction prevalence were reported as proportions. One-way ANOVA and independent t-tests were used for bivariate analysis to look at variations in mean BSMAS scores among different subgroups. The linear relationship between social media addiction and sleep quality was evaluated using Pearson's correlation coefficient. After controlling for potential confounders, multivariable linear regression was utilized to find predictors of social media addiction. Statistical significance was defined as a p-value <0.05.

## Observation and Results:

## Participant characteristics:

With 720 completed and valid for analyses out of the 750 distributed questionnaires, a 96 percent response rate was obtained. With participants ranging in age from 18 to 25, the mean age was 20.7 ± 1.9. 282 men (39.2 percent) and 438 women (60.8 percent) made up the study sample. Of the participants, the majority lived in hostels (55.6%), with the remainder being day scholars. 32 percent of students were pursuing undergraduate degrees in the arts, 28 percent were pursuing degrees in commerce and 26 percent were pursuing degrees in science. The remaining students were enrolled in professional courses.

## Prevalence of social media addiction:

According to the results of the Bergen Social Media Addiction Scale (BSMAS), 132 out of 720 students (18.3%) satisfied the requirements for social media addiction (score ≥24). For the whole sample, the average BSMAS score was 19.4 ± 5.6. Compared to men (15.2 percent), women (20.3 percent) had a significantly higher prevalence of addiction (p = 0.028).

## Social media usage patterns:

Platform Preference: In terms of social media usage, WhatsApp accounted for 92 percent, Instagram for 84 percent and Snapchat for 41 percent. There was a noticeable decrease in Facebook usage (18.9%).

## Duration of daily use:

[1] Less than 1 hour: 18.6%

[2] 1-3 hours: 52.9%

[3] More than 3 hours: 28.5%

## Behavioural observations:

[1] 36.1% of respondents said they checked social media as soon as they woke up.

[2] After extended use, 30.6% of people reported eye burning or watering.

[3] 19.2 percent reported that they use social media even during lectures or lectures at school.

[4] 22.5% had stayed up all night at least once using social media.

[5] Because there were fewer likes or comments, 7.8% of respondents said they felt emotionally impacted (in a sad or anxious way).

## Sleep quality assessment:

466 participants (64.7%) were categorized as having poor sleep quality (PSQI score >5) using the Pittsburgh Sleep Quality Index (PSQI). Participants' average global PSQI score was 7 points 8 ± 2 points 6.

## The most commonly affected sleep components were:

[1] Sleep latency: 53.2% reported moderate-to-severe difficulty falling asleep.

[2] Sleep duration: 41.5% slept fewer than 6 hours on average.

[3] Daytime dysfunction: 48.3% experienced fatigue or difficulty concentrating during the day.

[4] Use of sleeping medication: 5.1% reported occasional or frequent use.

## Association between social media addiction and sleep quality:

[1] Higher social media addiction scores were linked to lower sleep quality, according to a strong positive correlation found between BSMAS and PSQI scores (Pearson's r = 0.627, p < 0.001).

[2] A mean PSQI score of 9.3 ± 2.4 was significantly higher for students with social media addiction than for those without (6.9 ± 2.1, p < 0.001).

[3] The mean PSQI scores of individuals who used social media for more than three hours a day were lower (8.7 ± 2.3) than those who used it for less than an hour (6.3 ± 2.5, p < 0.001).

[4] Snapchat use was substantially linked to worse sleep latency (p = 0.009) and higher BSMAS scores (p = 0.001).

## Multivariate regression analysis:

Multivariable linear regression was performed to identify independent predictors of high BSMAS scores (Table 1). The model's adjusted R2 value was 0.61, meaning that the independent variables included in the model could account for about 61% of the variation in social media addiction scores.

## Discussion:

This cross-sectional study's findings revealed that nearly one in five college students in Chhindwara City met the criteria for social media addiction and over 64% of them had poor sleep quality. There is growing concern about the psychological and physical effects of youths' excessive digital use, as evidenced by the strong positive correlation between social media addiction (as measured by BSMAS) and poor sleep quality (as measured by PSQI). Results from comparable settings in India are in line with the study's findings regarding the prevalence of social media addiction (18.3%). As an illustration, Siddharthan *et al.* (2024) used the same diagnostic scale (BSMAS) to report a prevalence of 17.7 percent among medical students [11]. However, international studies have found a significantly higher prevalence: Setyowati *et al.* (2023) found that 76 percent of Indonesian nursing students had its [12] and Sserunkuuma *et al.* (2023) discovered that 74.3 percent of Ugandan medical students suffer from addiction [13]. Differences in sociocultural settings, academic demands and the availability of internet-enabled devices could all be contributing factors to these discrepancies. Sümen and Evgin (2021) found that female gender was a significant predictor of social media addiction. They attributed this to appearance-based content consumption, emotional regulation and social comparison behaviors, which are frequently more common among young women [5]. Furthermore, studies have indicated that females are more likely to engage with platforms that prioritize visual and interpersonal interaction, such as Instagram and Snapchat, which may make them more susceptible to addiction [10]. Students who used social media within 30 minutes of waking up had higher BSMAS scores, making early morning social media use another powerful predictor. The psychological idea of Fear of Missing Out (FoMO) is in line with this; it has been connected to obsessive checking habits and anxiety about the activities of peers or popular content [7].

Przybylski *et al.* (2013) found that people with high FoMO scores were much more likely to use social media frequently and intrusively, particularly in the morning [7]. Overuse of screens, especially social media, for longer than three hours a day has also been strongly linked to addiction and sleep problems. According to Bhargava and Velasquez (2021), algorithm-driven platforms in the attention economy take advantage of user behaviour to increase engagement, which frequently results in obsessive use [14]. Sleep disturbances are caused by the continuous scroll function and customized content feeds, which increase screen time and postpone bedtime rituals. Higher levels of addiction were found to be associated with worse sleep quality. The PSQI elements that were most impacted were daytime dysfunction, sleep duration and latency. This aligns with earlier research conducted by Levenson *et al.* (2016), Alsulami *et al.* (2019), which showed that social media . use at night is linked to fragmented sleep, delayed sleep onset and lower melatonin levels [4, 9]. Addiction scores were also substantially correlated with Snapchat use. Time spent online and psychological investment may be increased by the platform's design, particularly by elements like disappearing messages and Snap streaks. An investigation by Meshi *et al.* (2020) discovered that, perhaps as a result of the app's game-like rewards system, Snapchat users exhibited higher levels of compulsive behaviour than Facebook users [15]. Addiction scores were also noticeably higher for participants who reported burning or watering of the eyes after extended screen time. This lends credence to the existence of Digital Eye Strain, a condition that is now widely recognized among people who use screens a lot. Warad *et al.* (2022) demonstrated that prolonged, uninterrupted use of social media leads to ocular symptoms, which can interfere with sleep and exacerbate psychological discomfort [16]. Additionally, the study demonstrates that social media use and sleep quality are correlated in both directions. People who have trouble sleeping might use social media in the middle of the night to find company or distraction, which feeds a vicious cycle. Changing and associates. 2015 discovered that using light-emitting devices right before bed decreased melatonin levels and impacted sleep efficiency and alertness the following day [6]. This study has limitations despite its advantages, which include a sizable sample size, the use of validated instruments and sound statistical techniques. It cannot prove causation because it is cross-sectional. Recall or social desirability bias could affect self-reported data. The lack of assessment of variables like caffeine consumption, physical activity, academic workload and mental health status may have distorted the relationships that were found. Lastly, because of cultural and infrastructure disparities, the findings from Chhindwara might not apply to other areas.

## Conclusion:

We found a strong correlation between poor sleep outcomes and heavy social media use, especially extended daily use, early morning checking and participation on visually stimulating platforms like Snapchat. Data shows the critical need for behaviour modification techniques and awareness campaigns aimed at changing young people's digital habits. Unrestrained social media use is compromising sleep quality, which is essential for emotional control, academic achievement and general health. Promoting students' academic achievement and well-being requires addressing this modifiable risk factor.

## Recommendations:

Higher education institutions should set up structured programs to instruct students on the risks of excessive digital use, responsible social media use and the importance of screen-free time before bed. Awareness workshops should promote healthy sleep practices like consistent sleep schedules, avoiding screens at night and creating a sleep-friendly environment. Frequent assessment of digital addiction and sleep quality with validated tools (e.g. G. A. ought to be included in campus health exams or wellness clinics (BSMAS, PSQI). Academic establishments ought to consider introducing digital detox initiatives (e.g. G. A. "no-phone hours" or technologically-free zones) and promote in-person communication through extracurricular activities.

## Funding:

Nil

## Figures and Tables

**Table 1:** Significant predictors of social media addiction

**Variable**	**βCoefficient**	**95% CI**	**p-value**
Female gender	0.74	0.22-1.26	0.005
Use of social media within 30 minutes of waking	1.21	0.63-1.80	<0.001
Daily use >3 hours	1.35	0.81-1.89	<0.001
Use of Snapchat	0.92	0.33-1.52	0.003
Eye discomfort (burning/watering)	0.78	0.12-1.44	0.02
Poor subjective sleep quality (PSQI Component 1)	1.04	0.55-1.53	<0.001
